# Supplementary material for: The Prevalence of Multidrug-Resistant Enterobacteriaceae among Neonates in Kuwait
Source: Diagnostics (Basel). 2023 Apr 21;13(8):1505. doi: 10.3390/diagnostics13081505 (PMC10137823; doi:10.3390/diagnostics13081505)
Supplement: Supplementary file 1 [file diagnostics-13-01505-s001.zip › diagnostics-2308784-supplementary.pdf]

Supplementary

S.1 Research consent form

**1. Title of research**
The Prevalence of Multi Drug Resistance *Enterobacteriaceae* among Neonates at Farwaniya Hospital in Kuwait.
**2. Principal Investigator**
Dr. Wadha AlFouzan
**3. Why are we inviting you to join this research?**
Information of the neonate is required in research.
**4. What should you know about this research?**
• We will explain the research to you<br>• Whether or not you join is your decision (you can accept or refuse no matter who is inviting you to participate)<br>• Please feel free to ask questions or mention concerns before deciding, or during or after the research<br>• You can say yes but change your mind later<br>• We will not hold your decision against you
**5. Who can you talk to?**
If you have questions or concerns, or if you think the research has hurt you, talk to the research team at:<br>[Dr Wadha Alfouzan, Farwanyia hospital, Kuwait]<br>Telephone 99099222 (Sunday to Thursday 7.00 am to 3.00 pm) or E-mail: Alfouzan.w@hsc.edu.kw<br>If you have questions about your rights as a volunteer, or you want to talk to someone outside the research team, please contact:<br>• Ethical Research committee- MOH - Mr Alhassan Abdullah 90069869

6. Why are we doing the research?
This research will provide information on the status of *Enterobacteriaceae* resistance among neonates in Kuwait.
7. How long will the research take?
1 year
8. How many people will take part?
50 mothers and 50 neonates.
9. What happens if you take part?
If you agree to join, we will ask you to do the following:<br>Two rectal swab will be collected, one from you (the mother) and the other from neonate within 24 hours of delivery.<br>During your involvement in this research, a member of the research team will collect information including nationality, age, antibiotics consumed and the reason for consuming antibiotics during pregnancy.
12. What happens to information about you?
We will make efforts to secure information about you. This includes using a code to identify you in our records instead of using your name. We will not identify you personally in any reports or publications about this research.<br>We cannot guarantee complete secrecy, but we will limit access to information about you. Only people who have a need to review information will have access. These people might include:<br>• Members of the research team whose work is related to the research or to protecting your rights and safety
13. What if you don't want to join?
You can say no and we will not hold it against you.

Signature Page for Guardian of the baby

Volunteer

I voluntarily agree to join the research described in this form.

____
Printed Name of Volunteer

____
Signature of Volunteer Date

Person Obtaining Consent

I document that:

- *I (or another member of the research team) have fully explained this research to the volunteer.*
- *I have personally evaluated the volunteer's understanding of the research and obtained their voluntary agreement.*

Dr. Wadha AlFouzan
Printed Name of Person Obtaining Consent

____
Signature of Person Obtaining Consent Date

Witness (if applicable)

I document that the information in this form (and any other written information) was accurately explained to the volunteer, who appears to have understood and freely given Consent to join the research.

____
Printed Name of Witness

____
Signature of Witness Date

Signatures: Adult Unable to Consent
Legally Authorized Representative
I voluntarily agree for the person named below to join the research described in this form.
Printed Name of Volunteer
Printed Name of Representative Relationship
Signature of Representative Date
Person Obtaining Consent
I document that:<br>• *I (or another member of the research team) have fully explained this research to the representative.*<br>• *I have personally evaluated the representative's understanding of the research and obtained their voluntary agreement.*
Printed Name of Person Obtaining Consent
Signature of Person Obtaining Consent Date
Witness (if applicable)
I document that the information in this form (and any other written information) was accurately explained to the representative, who appears to have understood and freely given consent.
Printed Name of Witness
Signature of Witness Date

S.2 List of primers used for PCRs and sequence analysis

Antibiotic	Target gene	Sequence	Size of amplified DNA (bp)	Reference
**1st, 2nd and 3rd generation cephalosporin**	*bla-TEM*	F: GCGGAACCCCTATTTG<br>R:TCTAAAGTATATATGAGTAAACTTGGTCTGAC	964	[25]
	bla-SHV	F: TTCGCCTGTGTATTATCTCCCTG<br>R: TTAGCGTTGCCAGTGYTCG	854	[25]
	bla-CTX-M	F: ATGTGCAGYACCAGTAARGTKATGGC<br>R:TGGGTRAARTARGTSACCAGAAYCAGCGG	593	[25]
**Beta lactamase inhibitors**	*bla-TEM*	F: TAACCCTGGTAAATGCTTCA<br>R: CAATCTAAAGTATATATGAG	940	[13]
	bla-SHV	F: ATGCGTTWTDTTCGCCKGTG<br>R: TTAGCGTTGCCAGTGCTCGAT	861	[13]
	bla-OXA-1	F: CCATTATTTGAAGGAACTGA<br>R: TAGTGTGTTTAGAATGGTG	720	[13]
	bla-OXA-2	F: CCTGCATCGACATTCAAGATA<br>R: CTCAACCCATCCTACCCACCA	460	[13]
	bla-OXA-10	F: GCCATGAAAACATTTGCCGC<br>R: GCCACCAATGATGCCCTCAC	801	[13]
**Carbapenem**	*bla-IMP*	F: GGAATAGAGTGGCTTAAYTCTC<br>R: GGTTTAAYAAAACAACCACC	232	[26]
	bla-VIM	F: GATGGTGTTTGGTCGCAT<br>R: CGAATGCGCAGCACCAG	390	[26]
	bla-OXA-48	F: GCGTGGTTAAGGATGAACA<br>R: CATCAAGTTCAACCCAACC	438	[26]
	bla-GIM	F: TCGACACACCTTGGTCTGA<br>R: AACTTCCAACTTTGCCATG	477	[26]
	bla-NDM	F: GGTTTGGCGATCTGGTTTT<br>R: CGGAATGGCTCATCACGAT	621	[26]
	bla-KPC	F: CGTCTAGTTCTGCTGTCTT<br>R: CTTGTCATCCTTGTTAGGC	798	[26]

S.2 List of primers used for PCRs and sequence analysis (continued).

Antibiotic	Target gene	Sequence	Size of amplified DNA (bp)	Reference
**Folate pathway inhibitors**	*Sul 1*	F: TGGTGACGGTGTTCGGCATT<br>R: GCGAAGGTTTCCGAGAAGGT	790	[21]
	Sul 2	F: CGGCATCGTCAACATAACCT<br>R: TGTGCGGATGAAGTCAGCTC	721	[21]
	Sul 3	F: CAACGGAAGTGGGCGTTGTGGA<br>R:GCTGCACCAATTCGCTGAACG	244	[21]
	dfr1	F: TGGTAGCTATATCGAAGAATGGAG<br>R: TATGTTAGAGGCGAAGTCTTGGGT	425	[20]
	dfr 5	F: AGCTACTCTTTAAAGCCTTGACGT<br>R: GTGTTGCTCAAAAACAACTTC	341	[20]
	dfr7& 17	F: ACATTTGACTCTATGGGTGTTCTT<br>R: AAAACTGTTCAAAAACCAAATTGA	280	[20]
**Fluoroquinolones**	*qnrA*	F: GATAAAGTTTTTCAGCAAGAGG<br>R: ATCCAGATCGGCAAAGGTTA	543	[28]
	qnrB	F: GGCATTGAAATTCGCCACTG<br>R: TTTGCTGCTCGCCAGTCGA	360	[28]
	qnrS	F: TGGAAACCTACAATCATACA<br>R: TGCAATTTTGATACCTGATG	599	[28]
	aac(6')-Ib-cr	F: ATG ACT GAG CAT GAC CTT GC<br>R: TTA GGC ATC ACT GCG TGT TC	519	[28]
	qepA	F: AAC TGC TTG AGC CCG TAG AT<br>R: CGT GTT GCT GGA GTT CTT CC	189	[28]
	gyrA	F: AAA TCT GCC CGT GTC GTT GGT<br>R: GCC ATA CCT ACG GCG ATA CC	343	[28]
	parC	F: ATG TAC GTG ATC ATG GAC CG<br>R: ATT CGG TGT AAC GCA TCG CC	300	[28]
**Tigecycline**	*tetX1*	F: CGA AAA ATG TTG CTT GGC AGC TT<br>R: AGT TGT TGA ACG AAT TAA CTC C	486	[29]
	tetX2	F: CGG GAT GTC CAA GGT AAG AAA A<br>R: TGA CAA CGT CGT ATG AAT CAA	343	[29]
	tetX3	F: GAC ACT TGA TCT GCA CAG GGA TT<br>R: CCC TAC AAA AGA TGA TGT CAA AC	685	[29]
	tetX4	F: CTG ATT CGT GTG ACA TCA TCT TTT G<br>R: GTT AAA TTT CCC ATT GGT CAG ATT A	204	[29]
	tetX5	F: GGT ATC AAC ATT TCA ATG CTT G<br>R: CGA TTC GTC CTG CGT ATC TTT TG	256	[29]

S.2 List of primers used for PCRs and sequence analysis (continued).

Antibiotic	Target gene	Sequence	Size of amplified DNA (bp)	Reference
**Aminoglycosides**	*aac (3′)-II*	F: ATATCGCGATGCATACGCGG<br>R: GACGGCCTCTAACCGGAAGG	877	[27]
	aac (6′)-Ib	F: TTGCGATGCTCTATGAGTGGCTA<br>R: CTCGAATGCCTGGCGTGTTT	*472*	[27]
	aac (6′)-II	F: CGACCATTTCATGTCC<br>R: GAAGGCTTGTCGTGTTT	542	[27]
	ant(3″) -I	F: CATCATGAGGGAAGCGGTG<br>R: GACTACCTTGGTGATCTCG	*787*	[27]
	aph(3′)-VI	F: ATGGAATTGCCCAATATTATT<br>R: TCAATTCAATTCATCAAGTTT	780	[27]
	armA	F: CCGAAATGACAGTTCCTATC<br>R: GAAAATGAGTGCCTTGGAGG	*846*	[27]
	rmtB	F: ATGAACATCAACGATGCCCTC<br>R: CCTTCTGATTGGCTTATCCA	769	[27]
